# Stereotactic Radiotherapy and Particle Therapy for Pancreatic Cancer

**DOI:** 10.3390/cancers10030075

**Published:** 2018-03-16

**Authors:** Sweet Ping Ng, Joseph M. Herman

**Affiliations:** Department of Radiation Oncology, The University of Texas MD Anderson Cancer Center, 1515 Holcombe Blvd, Houston, TX 77030, USA; spng@mdanderson.org

**Keywords:** radiotherapy, stereotactic, proton, carbon, pancreatic cancer

## Abstract

Pancreatic cancer is a devastating disease with poor survival outcomes. Recent studies have shown that the addition of radiotherapy to chemotherapy in the setting of locally advanced pancreatic cancer did not improve overall survival outcome. These studies commonly utilize conventional radiotherapy treatment fractionation and technique (typically 3-D conformal radiotherapy or intensity modulated radiotherapy). Although no clear benefit in overall survival was demonstrated in those studies, those who received radiotherapy did have a clear benefit in terms of local control. Therefore, there is increasing interest in exploring different techniques and/or modality of radiotherapy and dose/fractionation. Stereotactic radiotherapy, which employs a hypofractionated regimen, has the potential advantage of delivering a high dose of radiation to the tumor in a short period of time (typically over 5 days) with minimal dose to the surrounding normal structures. Particle therapy such as proton and carbon ion therapy are being explored as potential radiation modality that could cause greater biological damage to the tumor compared to photon treatment, with rapid dose falloff resulting in minimal to no dose to adjacent structures. This review will discuss the current literature and emerging roles of stereotactic radiotherapy and particle therapy in pancreatic cancer.

## 1. Introduction

Pancreatic cancer, being the third leading cause of cancer-related deaths in the United States, is a devastating disease with an estimated 5-year overall survival of only 6% [1]. Although surgery provides patients with the best chance of achieving a cure, the majority of patients tend to present late with advanced stage of disease given the lack of symptoms and no effective screening tests available, as yet. The role of radiotherapy in the definitive management of pancreatic cancer has remained debated. The recent LAP07 study showed no overall survival with chemotherapy alone, as compared to chemoradiotherapy after completion of induction chemotherapy in patients with locally advanced pancreatic cancer (LAPC); however, the study did demonstrate that those who received radiotherapy had significantly improved local control with minimal increase in treatment-related toxicity [2]. Although local control of the disease did not translate to improvement in overall survival in the LAP07 study, there is evidence that in a subgroup of patients, local control could potentially translate to improvement in survival. Specifically, a John Hopkins rapid autopsy study indicated that approximately 1 in 3 patients died of local disease only [3]. As the LAP07 study employed conventional fractionation that were delivered over 6 weeks, there is speculation that some patients may have developed metastatic disease during this period of time when they were not on optimal systemic treatment. Therefore, there is increasing interest in exploring other radiotherapy options, particularly hypofractionated regimens and particle therapy, to maximize local therapy to the tumor whilst limiting patients’ time off effective systemic treatment. Here, we review the literature and highlight the emerging role of stereotactic body radiotherapy (SBRT) and particle therapy in the treatment of pancreatic cancer.

## 2. Stereotactic Body Radiotherapy (SBRT) for Locally Advanced Pancreatic Cancer

SBRT is an advanced technique of radiation planning and delivery where it delivers a highly conformal dose to the target with very steep dose gradient falloff at the edge of the target. This technique enables highly precise delivery of a high dose of radiation to the target in 1–5 fractions. As SBRT employs a hypofractionated regimen, particular care is required during planning and delivery because any geographic miss of the target could be a calamity, not only because the tumor was not adequately treated but also because the adjacent surrounding normal structures may receive higher than intended doses, thereby increasing the risk of toxicity. In a patient with pancreatic cancer, the adjacent normal structures include the duodenum, stomach and/or small bowel. Exceeding dose tolerance to these structures could cause gastrointestinal perforation and/or haemorrhage which can be fatal. Therefore, when considering SBRT, patient selection and adequate image guidance is paramount. 

In the past decade, there is increasing interest in utilizing SBRT when treating patients with pancreatic cancer. SBRT allows for the delivery of higher doses to the tumor and as most regimens are 5 fractions or less, patients only have to be off systemic treatment for a short period of time, as compared to conventional dose/fractionation regimens, where patients may be without adequate systemic therapy for up to 6 weeks. 

Table 1 summarizes the current literature. As depicted in Table 1, most studies demonstrated that local control with SBRT was approximately 80% at 1 year after treatment. However, survival rates remained poor as these patients were predominantly dying of distant disease. Hence, this provides further emphasis that these patients should have only limited time without systemic treatment.

### 2.1. Radiobiological Reasoning for SBRT

Various institutional and phase II study dose/fractionation has been reported. The current dose required to ablate the tumor has remained unknown. As the surrounding bowel structures are relatively radiosensitive, the total dose that can be delivered to the tumor is currently limited by the tolerance dose of adjacent normal structures. 

The effect of escalated dose to the tumor on patient outcomes have been demonstrated by the MD Anderson group. Krishnan et al. [4] have reported, in a group of 200 patients with LAPC treated, using fractionated intensity modulated radiotherapy (IMRT), those who received the biological equivalent dose (BED) of >70 Gy had superior overall survival and locoregional relapse free survival, as compared to those who had BED ≤70 Gy, regardless of tumor size or frequency of surgical resection. Only 1 patient developed Grade 3 acute toxicity (diarrhea) in this analysis. 

As SBRT is a highly conformal treatment with rapid dose falloff beyond the target, hypofractionation has been employed to deliver high dose per fraction (>2 Gy) to the tumor with limited dose to the normal structures, thereby maximizing the therapeutic ratio. Furthermore, delivering an intended dose of treatment in a shorter period of time was thought to have greater biological kill in a tumor that is theorized to have a low alpha/beta ratio of 3 [5]. Fractionated SBRT is preferred due to better tolerability of treatment, reduced late effect toxicity, and allowing time for reoxygenation of hypoxic tumor cells and redistribution of resistant tumor cells into a more radiosensitive cell cycle phase [5].

### 2.2. Dose/Fractionation and Toxicities

The optimal dose/fractionation for SBRT has yet to be established. Initial studies have shown that fractionated (3–5 fractions) regimens are, in general, better tolerated than single fraction treatments. Although the safety of single fraction SBRT was initially established by the Stanford group in 6 patients with locally advanced pancreatic cancer (LAPC), subsequent larger trials showed excellent efficacy but with high rates of late toxicity (20% rate of Grade ≥2 late toxicity) [6,7]. Subsequently, a multi-institutional phase II study reported by Herman et al. [8] demonstrated equivalent efficacy with 1-year freedom from local progression of 78% with acceptable toxicity profile (11% rate of Grade ≥2 late toxicity). Other studies using fractionated regimens, as described in Table 1, have similar late toxicity profile.

Given the location of the pancreas to the small bowel, stomach and biliary structures, the most common subacute and late toxicities include gastrointestinal ulcer, which may result in perforation and/or bleeding, and fibrosis of the region causing biliary or duodenal stricture leading to obstruction. Although the rates of Grade ≥3 toxicities are low, these radiation-induced toxicities can potentially cause significant morbidity and/or fatal. Therefore, the dose tolerance of normal structures must be respected during treatment planning and delivery, with adequate daily on-board imaging to identify where these structures are located daily in proximity to the tumor.

### 2.3. Reirradiation

Although distant relapse remained the primary cause of pancreatic cancer-related death, the local progression of disease contributed to approximately 30% of pancreatic cancer-related deaths [3,18]. Standard of care is to evaluate these patients for further surgery. However, this subgroup of patients is frequently non-surgical candidates due to patient-related factors (age, comorbidities, performance status) and/or treatment-related factors (technically unresectable). In patients with limited distant disease burden, radiotherapy can be re-considered to delay disease progression that can potentially cause significant morbidity such as pain and/or obstruction [18,19].

**Figure 1:**
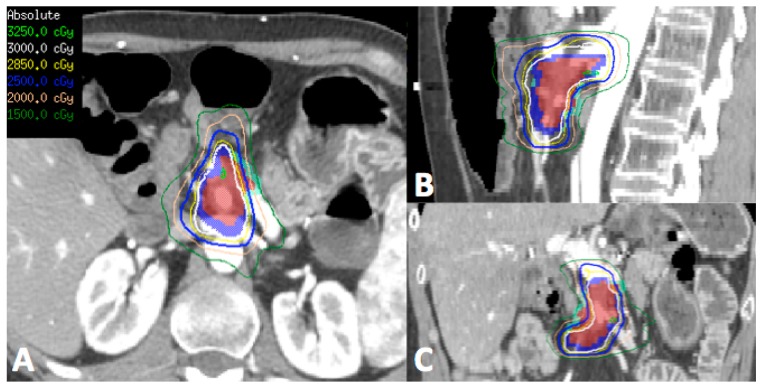
Representative transverse (**A**), sagittal (**B**) and coronal (**C**) images of the reirradiation treatment plan using stereotactic body radiotherapy (SBRT) for a patient who had previous 3D conformal radiotherapy to the region 5 years ago to 50.4 Gy in 28 fractions. (Red—gross tumor volume, Dark blue—target for 30 Gy, Light blue—target for 25 Gy due to proximity to surrounding bowel and stomach). Patient tolerated treatment well with Grade 1 nausea and was alive 12 months post-treatment with no significant late toxicity.

In patients who have had previous radiotherapy, it is imperative to obtain previous treatment plans to assess dose in relation to surrounding normal structures, in particular the duodenum, small bowel and stomach (Figure 1). Reirradiation in this setting could potentially cause significant late effects, depending on the cumulative doses to the normal structures. SBRT is becoming increasingly accepted when patients are considered for reirradiation in the attempt to maximize dose to the tumor whilst limiting the dose to surrounding normal organs thereby reducing the risk of significant late effects.

SBRT is potentially a feasible and safe option in patients with local recurrence. Table 2 summarizes the studies where SBRT was utilized in the setting of local recurrence with the majority of publications on patients who received previous radiotherapy. As this group of patients is small, the evidence is limited to retrospective studies alone. Nevertheless, from Table 2, SBRT can achieve good local control (1-year freedom from local progression of 62–91%). Although the incidence of grade 3 or higher late toxicity is rare, great care should be taken at treatment planning and delivery with regards to the stomach and bowel dose as gastrointestinal toxicity may cause significant morbidity to this group of patients with limited life expectancy.

## 3. Particle Therapy for Pancreatic Cancer

### 3.1. Proton Therapy

**Figure 2:**
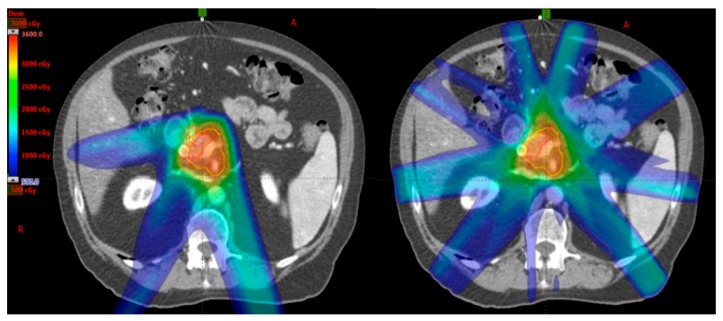
An example of proton (**Left**) and photon (**Right**) SBRT plans on the same patient. Note that there is less low dose scatter in the proton plan due to the inherent physical properties of proton particles compared to photon.

The proton beam, a particle therapy, has the benefit of delivering dose to the target with no exit dose in the beam path (Figure 2); thereby potentially reducing dose to the normal tissues within the exit beam path [25]. This, theoretically, may reduce both acute and late toxicities of treatment. In addition, with minimal dose in the exit beam path, proton therapy opens the door to possible dose escalation studies. As proton therapy is a relatively new player in the clinical radiation oncology domain, the evidence for the use in pancreatic cancer remains limited. Table 3 summarizes the current literature for particle therapy in pancreatic cancer.

A dosimetric study comparing IMRT and proton plans in 13 patients with unresectable pancreatic cancer, planned to 55 Gy in 25 fractions, by Thompson et al. [26] demonstrated that proton plans, utilizing either passive scattering or pencil beam scanning, yielded significantly lower doses to the stomach, duodenum and small bowel in the intermediate to low dose regions (defined as volume receiving at least 20 Gy) compared to IMRT. However, in the intermediate to high dose regions (defined as volume receiving at least 45 Gy), proton plans had significantly higher dose to those structures compared to IMRT. However, proton plans did yield significant reduction in mean liver (50% reduction) and kidney (18% reduction) doses. The biological significance of reduced dose to the low dose regions remained to be investigated.

As proton is a charged particle, it has a higher linear energy transfer [27]. Therefore, proton therapy can potentially deliver higher relative biological effectiveness than photon therapy [27]. In theory, proton therapy could result in greater cell killing than photon therapy given the same dose/fractionation. Although this is a desirable effect on cancer cells, this may also indicate that greater care is required when delivering proton therapy as a small increase in dose to normal structures could potentially translate to greater risk of late toxicity compared to photon therapy. The exact ‘conversion’ of proton therapy dose effect to photon therapy equivalence remained under investigation. As image guidance in proton therapy is being developed and implemented, careful planning and motion management should be taken when delivering proton therapy to a moving target such as the pancreas.

### 3.2. Carbon Ion Therapy

It has been theorized that heavy ion particle therapy, such as carbon ion therapy, provides greater biological effect due to even higher linear energy transfer of the particle which translates to greater relatively biological effectiveness. Currently, there are no carbon ion facilities in North America. Our Japanese colleagues, Shinoto et al. [28] first reported on the use of carbon ion therapy in patients with pancreatic cancer. In this phase I dose-escalation trial, a cohort of 26 patients were treated with pre-operative carbon ion therapy in 8 fractions over 2 weeks (total dose: 30 Gray equivalents [GyE] to 36.8 GyE). Twenty-one patients had surgery, with 19 patients achieving an R0 resection. There was no local recurrence reported in this cohort and only 2 patients developed regional recurrence. The 5-year overall survival for this cohort was 42%. Only one patient developed late Grade 4 toxicity with portal vein stenosis and deranged liver function. 

In a cohort of patients with locally advanced pancreatic cancer treated with a dose-escalation protocol up to 55.2 GyE, Shinoto et al. [29] reported the treatment was well-tolerated and that those who received ≥45.6 GyE had overall better clinical outcomes in terms of local disease control and overall survival than those who received lower doses.

## 4. Conclusions

Overall, SBRT and proton therapy are emerging novel radiotherapy techniques/modalities that could potentially revolutionize treatment for patients with pancreatic cancer. These techniques allow the gain of local control benefit with minimal toxicity in patients with LAPC whilst minimizing their time away from optimal effective systemic treatment. With advancements in imaging and radiation treatment planning, current efforts to improve clinical outcomes in those with LAPC in the realm of radiation therapy includes dose-escalation trials to maximize local and/or regional control and MR-guided radiation therapy.

## Figures and Tables

**Table 1:** Summary of studies reporting on outcomes of patients treated with Stereotactic Body Radiotherapy (SBRT).

Study	Nature	Number of Patients	Dose/Fractionation	Outcomes	Toxicity (≥Grade 3)
Chang et al., 2009 [9]	Retrospective	77, unresectable (included metastatic patients)	25 Gy/1 fraction	1-year FFLP: 84%, 1-year PFS: 9%. 1 year OS: 21%	Acute: Gastric ulcer (1), Late: Gastric ulcer (3), duodenal stricture (1), biliary stricture (2)
Chuong et al., 2013 [10]	Retrospective	73, LAPC and borderline	25 Gy/5 fractions	1-year PFS: 42.8% (borderline), 41% (LAPC), 1-year OS: 72.2% (borderline), 68.1% (LAPC)	Late: GI bleeding (3), anorexia (1)
Comito et al., 2017 [11]	Phase II, single institution	45, LAPC	45 Gy/6 fractions	2-year FFLP: 90%, Median OS: 23.5 months	None
Gurka et al., 2017 [12]	Retrospective	38, LAPC and borderline	25–30 Gy/5 fractions	6-month FFLP: 82%, Median PFS: 6.8 months, Median OS: 12.3 months	Late: Gastric outlet obstruction (1), biliary obstruction (1), GI bleeding (1—grade 5)
Herman et al., 2015 [8]	Phase II, multi-institutional	49, LAPC	33 Gy/5 fractions	1-year FFLP: 78%, 1-year PFS: 32%, 2-year PFS: 10%, 1-year OS: 59%, 2-year OS: 18%	Acute: Duodenal ulcer (1), elevated liver function tests (5), Late: fistula (1), ulcer (3)
Mahadevan et al., 2011 [13]	Retrospective	39, LAPC	24–36 Gy/3 fractions	Local control 85% (median follow up: 21 months), Median DFS: 15 months, Median OS: 20 months	Late: GI bleeding (2), gastric outlet obstruction (1)
Mellon et al., 2015 [14]	Retrospective	159 (110 borderline + 49 LAPC)	28–30 Gy/5 fractions	Median OS: 19.2 months (borderline), 15 months (LAPC)	Late: GI bleeding (6)
Pollom et al., 2014 [15]	Retrospective	167, unresectable	25 Gy/1 fraction (76), 25–45 Gy/5 fraction (91)	1-year OS: 33.1%	Acute: GI bleed (1), gastric ulcer (1), Late: Duodenal perforation (3), biliary stricture (1), gastric ulcer (4), GI bleed (1), duodenal ulcer (2), duodenal stricture (2)
Schellenberg et al., 2008 [6]	Phase II, single institution	16, LAPC	25 Gy/1 fraction	1-year OS: 50% Median OS: 11.4 months	Acute: gastric outlet obstruction (1), Late: duodenal perforation (1), duodenal stenosis (1)
Tozzi et al., 2013 [16]	Prospective, single institution	30, (21 LAPC + 9 recurrence post surgery)	45 Gy/6 fractions	1-year FFLP: 77%, 2-year FFLP: 77%, Median PFS: 8 months, 1-year OS: 47%	None
Rwigema et al., 2011 [17]	Retrospective, single institution	71 (40 LAPC, 11 recurrence post surgery, 8 with metastatic disease, 12 adjuvant treatment)	18–25 Gy/1–4 fractions	1-year FFLP: 48.5%, (73% in those who received ≥24 Gy vs 45% in those who had lower doses), 1-year OS: 41%, Median OS: 10.3 months	Acute: nausea (1), enteritis (1), gastroparesis (1), Late: None

LAPC: Locally advanced pancreatic cancer; FFLP: Freedom from local progression; PFS: Progression free survival; OS: Overall survival; DFS: Disease free survival.

**Table 2:** Summary of studies utilizing SBRT for treatment of local recurrence.

Study	Nature	Number of Patients	Dose/Fractionation	Outcomes	Toxicity (≥Grade 3)
Comito et al., 2017 [20]	Retrospective	31, after R0 surgery	45 Gy/6 fractions	1-year FFLP: 91%, 2-year FFLP: 82%, Median PFS: 9 months, Median OS: 18 months	None
Dagoglu et al., 2016 [21]	Retrospective	30, reirradiation	Median dose: 25 Gy (24–36), Median no of fractions: 5 (3–5)	1-year FFLP: 78%, 2-year FFLP: 78%, Median OS: 14 months	Acute: GI bleeding (1), Vomiting (1), pain (1), Late: Bowel obstruction (2)
Koong et al., 2017 [22]	Retrospective	23, reirradiation	9 patients had 25 Gy/1 fraction; 14 patients had 20–33 Gy/1–5 fractions	1-year FFLP: 81%, Median OS: 8.5 months	Acute: Gastric fistula (1), gastric ulcer (1)
Lominska et al., 2012 [23]	Retrospective	28, reirradiation	Median: 22.5 Gy (20–30), Median no of fractions: 3 (3–5)	1-year FFLP: 70%, Median OS: 5.9 months	Late: Bowel obstruction (1), gastric perforation (1)
Ryan et al., 2018 [24]	Retrospective	25 out of 51 patients received reirradiation, all patients had previous surgery	Median: 25 Gy No of fractions: 5 fractions	For reirradiated group, 1-year FFLP: 37%, Median PFS: 7 months, Median OS: 11 months	Acute: Bowel obstruction (1), Late: Bowel obstruction (2), GI bleeding (1)
Wild et al., 2013 [19]	Retrospective	18, reirradiation	Median dose: 25 Gy (20–27)/5 fractions	1-year FFLP: 62%, Median OS: 8.8 months	Late: Small bowel obstruction (1)

**Table 3:** Summary of particle therapy studies.

Study	Nature	Number of Patients	Dose/Fractionation, Concurrent Chemotherapy	Outcomes	Toxicity (≥Grade 3)
Hong et al., 2014 [30]	Prospective; Neoadjuvant (Proton)	50	25 Gy/5 fractions, Capecitabine	11 patients did not have surgery, Out of 48 patients: PFS: 10.4 months, OS: 17.3 months, 2-year OS: 42%, Out of 37 patients who had surgery: PFS: 14.5 months, OS: 27 months	Acute: colitis (1), chest wall pain (1)
Sachsman et al., 2014 [31]	Prospective; Definitive (Proton)	11	59.4 Gy/33 fractions, Capecitabine	2-year PFS: 14%, OS: 18.4 months, 2-year OS: 31%, 2-year FFLP: 69%	None
Terashima et al., 2012 [32]	Prospective; Definitive (Proton)	50	P1: 50 Gy/25 fractions (5), P2: 70.2 Gy/26 fractions (5), P3: 67.5 Gy/25 fractions (40), All with concurrent gemcitabine	Overall: 1-year PFS: 64.3%, 1-year OS: 76.8%, 1-year FFLP: 81.7%, P3 patients: 1-year PFS: 60.8%, 1-year OS: 78.8%, 1-year FFLP: 79.9%	P1 and P2:, Acute GI bleeding (1), P3: GI ulcer treated with medications (3); death from GI bleed (1)
Shinoto et al., 2013 [28]	Prospective, Phase I, neoadjuvant (Carbon)	21	30–36.8 GyE/5 fractions, No concurrent chemotherapy	No local recurrence, 1-year PFS: 40%, 1-year OS: 69%, 5-year OS: 42%, Median OS: 18.6 months	Acute: Liver abscess (1), Late: Deranged liver function due to portal vein stenosis (1)
Shinoto et al., 2016 [29]	Prospective, Phase I, LAPC (Carbon)	71	43.2–55.2 GyE/12 fractions, Gemcitabine	1-year OS: 73%, 2-year OS: 35%, Median OS: 19.6 months, Better outcomes in those who had ≥45.6 GyE	Acute (non-hematologic): Anorexia (6), GI bleed (1)
